# The relationship between psychological factors and pain in endometriosis

**DOI:** 10.1177/17455057261444146

**Published:** 2026-04-27

**Authors:** R. Favaloro, K. T. Hallam, P. Saunders

**Affiliations:** 1 Department of Psychology, RMIT University, Bundoora, VIC, Australia

**Keywords:** endometriosis, pain, self-efficacy, catastrophizing, hypervigilance

## Abstract

**Background:**

Endometriosis is a prevalent condition characterized by persistent pelvic pain, which leads to distress and reduced quality of life. Currently, there is limited research examining the psychological factors associated with the symptoms of endometriosis, including potential risks and protective factors. The fear-avoidance model was identified to be a potential model to describe how psychological factors may impact the pain-related symptoms of endometriosis, informing the role that hypervigilance, pain catastrophizing, and avoidance behaviors play in increasing pain and distress. Previous research exploring this model identified self-efficacy and functionality appreciation as potential factors in reducing pain and distress in individuals with endometriosis.

**Objectives:**

The present study explores the fear-avoidance model’s application to pain-related symptoms and health-related quality of life (HRQoL) in people with endometriosis, and the interaction between these variables to better understand the psychological processes linked to increased pain and HRQoL in individuals with endometriosis.

**Design:**

This study was cross-sectional observational design, with data collection and analysis conducted over the course of 6 months following ethics approval.

**Methods:**

A total of 332 participants (female = 94%, male = 2.7%, non-binary/third gender = 3.3%) aged between 18 and 57 (*M* = 31.14, SD = 7.93) completed an online questionnaire assessing self-efficacy, functionality satisfaction, pain hypervigilance, pain catastrophizing, sexual avoidance and functioning, pain avoidance and pain severity. Correlational analysis, multiple linear regression and moderation analysis were conducted.

**Results:**

Functionality appreciation and self-efficacy were both associated with decreased pain and increased HRQoL. Hypervigilance functioned as an enhancing moderator for the relationship between sexual avoidance and pain, while pain catastrophizing acted as an enhancing moderator for the relationship between pain and HRQoL.

**Conclusion:**

The overall findings of the present study revealed the expanded application of the fear-avoidance model, and its use in conceptualizing pain in endometriosis. This includes the role of functionality appreciation and self-efficacy as potential protective factors against increased pain in endometriosis. This study supports the development of interventions targeting hypervigilance, and pain catastrophizing as valuable strategies for pain management and improving HRQoL among individuals with endometriosis.

## Introduction

Endometriosis is a chronic gynecological condition where endometrial-like tissue develops outside the uterus.^1^ Impacting around 1 in 10 women of reproductive age, endometriosis is associated with persistent pain and significant functional impairment.^2–4^ The primary symptoms of endometriosis include chronic pelvic pain, menstrual pain (dysmenorrhea), pain during intercourse (dyspareunia), painful urination (dysuria), painful defecation (dyschezia), and infertility.^5,6^

The subjective health-related quality of life (HRQoL) of individuals with endometriosis is shaped by the interplay between physiological factors—particularly pain intensity and chronicity—and psychological and social influences. This biopsychosocial interaction encompasses experiences of pain, perceived control and powerlessness, emotional well-being, social support, and self-image, and is commonly assessed using measures such as the endometriosis health profile-30 (EHP-30)^7^ and the SF-36.^8^ In a large international study of over 900 women, De Graaff et al.^9^ demonstrated that endometriosis affected work functioning in 51% of participants and intimate relationships in 50%, alongside significant reductions across all eight SF-36 quality-of-life domains.^9^ Consistent with this, cross-sectional studies show that lower HRQoL is associated with greater pain severity, longer symptom duration, and higher overall symptom burden.^10^ Notably, in this study, the most pronounced impact of physical symptoms was observed in the EHP-30 subscale of control and powerlessness. Even among women with complex presentations, including deep infiltrating endometriosis, Yela et al.^11^ reported the greatest HRQoL impairments in social well-being, infertility-related concerns, and sexual functioning.^11^ Importantly, social support plays a critical role in shaping HRQoL outcomes.^12^

Anxiety and depression are particularly common in women with endometriosis, with prevalence rates ranging from 20% to 85%.^3,5,6,13^ While biomedical research on the HRQoL impacts of endometriosis has increased, literature on the psychological predictive and perpetuating factors is limited, leaving clinicians underprepared to understand the psychological factors, which may lead to increased pain, emotional distress, and reduced HRQoL in women experiencing endometriosis.^3,6^

In terms of psychological factors that impact chronic pain conditions, the fear-avoidance model has proven particularly helpful in understanding the experience and persistence of chronic pain symptoms.^14,15^ Developed in 1983,^14^ the fear-avoidance model poses that when pain is appraised as dangerous, attention becomes increasingly focused on bodily sensations and pain signals, and negative expectancies develop about movement and harm. These processes heighten fear and catastrophizing, leading to avoidance of activities. While this avoidance may be sensed as protective in the short term, over time this leads to hypervigilance and emotional distress. These then increase the experience of pain and functional impairment, and this process is believed to play a role in the transition to impairment associated with chronic pain.^16–18^

No studies to date have specifically investigated the fear-avoidance model in relation to endometriosis, however, research on related conditions sheds light on the potential utility of the model for this population. Thomtén and Linton^19^ have applied the fear-avoidance model to the study of dsypareunia (pain during intercourse), demonstrating similarities between dyspareunia and chronic pain conditions using this framework. They further highlight that unlike pain conditions, specific and important consequences of dyspareunia include additional emotional consequences and complexity around partners role. A more recent study^20^ of women with dyspareunia highlighted significantly higher scores on pain catastrophizing and anxiety than the control group. Furthermore, Thomtén et al.^21^ found fear avoidance alongside pain catastrophizing in women who report experiencing sexual pain similar to the level experienced by people with other chronic pain conditions. Notably, these fear-avoidance beliefs were positively correlated with pain intensity, indicating the greater the experience of pain, the stronger the fear avoidance. Importantly, endometriosis has been consistently associated with a decrease in sexual functioning due to the symptoms of dyspareunia.^22^ Shi et al.^23^ analyzed the effects of endometriosis on sexual functioning across six studies and found that women with endometriosis scored significantly lower across all domains of the female sexual function index (FSFI) compared to those without.

Rabinowitz et al.^24^ explored painful menstrual cramps, a common symptom of endometriosis, in a systematic review of catastrophizing and chronic cyclical pelvic pain. The review found a small but significant positive association between catastrophizing and pain ratings, suggesting that psychological interventions may benefit patients with cyclical pelvic pain.^24^ However, many studies in the review had small sample sizes and inconsistent psychometrics, limiting generalizability and the ability to assess confounding variables. Overall, the current literature provides limited research on the relationship between the fear-avoidance model and endometriosis, despite strong theoretical support.

The fear-avoidance model provides a valuable framework for understanding risk factors that exacerbate pain and distress in individuals with endometriosis. However, it is equally important to identify protective factors, such as self-efficacy and functionality appreciation, that may mitigate these effects and inform the development of more effective interventions. Self-efficacy, the confidence in one’s ability to complete a task to achieve a desired outcome, has been widely studied as a key psychological influence on chronic pain.^25,26^ In the context of endometriosis, a condition that includes significant levels of pain for many individuals and requires long-term treatment, numerous interactions with healthcare systems, and adaption to chronic symptoms over time, self-efficacy can act as a buffer against the deterioration of HRQoL.^10,27^ Bień et al.^27^ study using the EHP-30 found that self-efficacy was associated with higher HRQoL in areas such as pain, sense of control and powerlessness, self-assessment, and relationships with medical staff, the latter of which is important to highlight as people convinced of their self-efficacy are more likely to formulate expectations and actively corporate with medical professionals, potentially leading to greater satisfaction with treatment.^27^ Two key hypotheses explain this association: (1) self-efficacy influences the performance of pain-management actions, and (2) perceived self-efficacy affects how pain-related situations are managed.^26^ These hypotheses underline the role of self-efficacy in the fear-avoidance model, whereby individuals with low pain coping self-efficacy may avoid pain-related activities.^26^ Maxwell Slepian et al.^28^ explored the connection between the fear-avoidance model and positive psychological constructs like pain resilience and self-efficacy. They proposed expanding the fear-avoidance model to include these protective mechanisms, which may help break the cycle in the model. Although this idea requires further validation, growing research supports the relationship between self-efficacy and improved functioning and symptom management in individuals with endometriosis.^18,26,28^

Self-efficacy has been identified as an important factor in the management of chronic pain conditions, with higher levels linked to reduced lower pain severity, functional impairment, and distress.^23^ Similar patterns have been found within endometriosis populations. Facchin et al.^25^ examined predictors of psychological distress in endometriosis patients, including self-esteem, body esteem, and emotional self-efficacy. They found that individuals with greater self-efficacy experienced less distress from their symptoms, suggesting it as a promising target for future interventions.

Alleva et al.^29^ introduced the concept of functionality appreciation, defined as appreciating and respecting the body for its capabilities. This concept has gained importance in pain populations^30^ and is emerging as an important factor in endometriosis. Mills et al.^31^ conducted a qualitative study on women with endometriosis and their body perceptions, revealing three major themes: (1) feeling broken and inadequate, (2) feeling at war with the body, and (3) feeling alienated from the body. These themes reflect a sense of defectiveness, conflict, and alienation, emphasizing a lack of control in managing symptoms.^32^ Grano et al.’s^32^ study of 232 women with endometriosis found that functionality appreciation mediated the relationship between severity of dyspareunia and sexual distress, highlighting the important role of functional acknowledgment and appreciation in endometriosis.

### The present study

Endometriosis is a common, chronic condition with limited research on the psychological factors involved in its symptom management. Previous studies highlight the negative impact of endometriosis on the lives of those affected, showing how symptom severity affects HRQoL and mental health.^3,4,33,34^ Both qualitative and quantitative reports support these findings, though there are still gaps in endometriosis research. Limited theoretical work has been done with endometriosis populations, but studies on overlapping symptoms suggest the fear-avoidance model may help uncover psychological mechanisms, such as pain catastrophizing, hypervigilance, and avoidance behaviors.^20,21,35,36^ Conversely, psychological factors like self-efficacy and functionality appreciation are associated with symptom improvement in chronic pain literature.^26,30,31,37,38^

The present study explores the fear-avoidance model’s application to pain-related symptoms and HRQoL in people with endometriosis, and the interaction between these variables to better understand the psychological processes linked to increased pain and HRQoL in individuals with endometriosis. Based on previous literature, it was hypothesized that functionality appreciation is positively associated with self-efficacy. It was further hypothesized that the relationship between sexual avoidance and pain is moderated by pain catastrophizing and hypervigilance. Finally, it was hypothesized that the relationship between pain and HRQoL will be moderated by self-efficacy, functionality appreciation, hypervigilance, pain catastrophizing, and sexual functioning.

## Method

### Participants

The present study received ethics approval by the RMIT University Human Research Ethics Committee (approval number: 27655) as it meets the requirements of the National Statement on Ethical Conduct in Human Research.^39^ This study is a cross-sectional observational study that conforms to the Strengthening the Reporting of Observational Studies in Epidemiology (STROBE) statement (Supplement material).^40^ The study was conducted from May 2024 to November 2024, and the total duration of the study including data collection and analysis was over the course of 6 months following ethics approval. The sample consisted of 332 participants with a self-reported diagnosis of endometriosis by a medical professional aged between 18 and 57 (*M* = 31.14, SD = 7.93). When screening for diagnosis of endometriosis, participants were required to identify if their diagnosis was confirmed with laparoscopic surgery, and of the 332 participants diagnosed with endometriosis by a medical professional, 256 (77.1%) of participants were diagnosed through laparoscopic surgery. About 312 participants identified as female (94%), 9 as male (2.7%), and 11 as non-binary or third gender (3.3%). Most studies on endometriosis have only included samples of cis-gendered women (i.e., women who were assigned female at birth) often neglecting gender-diverse individuals.^33^ As endometriosis is a condition that effects the uterus, the inclusion of participants who did not identify as a woman was intentional to allow a richer sample.^16,41^ The majority of participants were born in Australia (30.72%) and varied in education level and relationship status (Table 1). Participants were included if they were over the age of 18 and proficient in English. Those who did not meet these criteria, give consent, or exited the survey early were excluded.

**Table 1.** Participant characteristics.

Demographics	*N* (%)	*M* (SD)
Age	—	31.14 (7.93)
Gender		
Male	9 (2.7)	—
Female	312 (94.0)	—
Non-binary/third gender	11 (3.3)	—
Relationship		
Single	102 (30.7)	—
In a relationship	123 (37.0)	—
Married	100 (30.1)	—
Education		
Less than high school	10 (3.0)	—
High school graduate	62 (18.7)	—
Some college	61 (18.4)	—
2-year degree	25 (7.5)	—
4-year degree	82 (24.7)	—
Professional degree	74 (22.3)	—
Doctorate	17 (5.1)	—
Employment status		
Unemployed	77 (23.2)	—
Casual	26 (7.8)	—
Part-time	59 (17.8)	—
Full-time	152 (45.8)	—
Seasonal contract	2 (0.6)	—
Other	16 (4.8)	—
Country of birth		
Oceania	105 (37.2)	—
North America	63 (22.3)	—
South America	2 (0.7)	—
Europe	24 (8.5)	—
Asia	7 (2.5)	—
Africa	81 (28.7)	—
Diagnosed by laparoscopic surgery		
Yes	256 (77.1)	—
No	73 (21.9)	—
Scales		
GSE	—	30.56 (5.16)
FAS	—	3.78 (.90)
PVAQ	—	52.85 (15.33)
PCS	—	27.91 (13.47)
FSFI	—	17.49 (9.00)
EHP-30	—	66.58 (17.07)
VAS	—	7.45 (2.14)

GSE: general self-efficacy scale; FAS: functionality appreciation scale; PVAQ: pain vigilance and awareness questionnaire; PCS: pain catastrophizing scale; FSFI: female sexual function index; EHP-30: endometriosis health profile-30; VAS: visual analog scale; SD: standard deviation.

Little’s MCAR test, which assesses whether data are missing completely at random, concluded that values were missing completely at random ( $\chi^{2}$ = 4168.78, *p* = 0.53). The expectation maximization method was utilized to impute missing values at the item level. The imputed dataset was then used to calculate the totals of each scale and conduct the remaining analyses. The recommended sample size was identified in the a priori power analysis, conducted with G-Power software. Assuming a small-to-medium interaction effect (*f*^2^ = 0.05), an alpha level of 0.05, and power of 0.80, a minimum sample of approximately 263 participants was required. The study exceeded the recommended sample size of 263.

### Materials

The Qualtrics software package (Qualtrics, Provo, UT) was utilized to administer six demographic questions (age, country of birth, gender, highest level of education, employment status, and relationship status), six questions regarding medical history (pregnancy, breastfeeding, diagnosis of endometriosis, previous hysterectomy, whether they had any prior children and current medication) and the following validated questionnaires assessing self-efficacy, functionality satisfaction, pain vigilance, pain catastrophizing, sexual avoidance and functioning, pain avoidance, and pain severity.

#### General self-efficacy scale

The general self-efficacy scale was developed by Schwarzer and Jerusalem (1995-English translation version) and is a 10-item self-report measure of self-efficacy over a 4-point Likert scale ranging from 1 (not at all true) to 4 (exactly true). The sum of the 10 items is calculated to create a total score. Total scores ranged from 10 to 40 with higher scores indicating higher self-efficacy.^42^ Cronbach’s internal consistency reliability coefficient (CICRC) within the current sample was α = 0.89, consistent with the original research.^42^

#### Functionality appreciation scale

The functionality appreciation scale is a seven-item self-report measure used to assess self-functionality satisfaction over a 5-point Likert scale ranging from 1 (strongly disagree) to 5 (strongly agree). The scores on all seven items were averaged, with higher scores indicating higher levels of functionality appreciation.^29^ CICRC within the current sample was α = 0.92.

#### Pain vigilance and awareness questionnaire

The pain vigilance and awareness questionnaire is a 16-item self-report measure used to assess attention to pain over a 6-point Likert scale ranging from 0 (never) to 5 (always). The score on all 16 items were summed, with items 8 and 16 being reversed scored. The total questionnaire scores range from 0 to 80 with higher scores indicating increased attention to pain.^43^ CICRC within the current sample was α = 0.93.

#### Pain catastrophizing scale

The pain catastrophizing scale is a 13-item measure used to assess the degree of pain catastrophizing over a 5-point Likert scale ranging from 0 (not at all) to 4 (always). These 13 items are categorized into 3 subscales: Rumination, Magnification, and Helplessness; however, only the total score was utilized in this study. The sum of all 13 items were calculated to create a total score. The total score ranges from 0 to 52 with higher scores indicating a greater degree of pain catastrophizing and a total score of >30 representing a clinically significant level of pain catastrophizing.^44,45^ CICRC within the current sample was α = 0.95.

#### Female sexual function index

The FSFI is a 19-item measure used to assess sexual avoidance and functioning over a 5-point Likert scale ranging from 1 (almost never or never) to 5 (almost always or always). The 19-items are categorized into 6 domains: Desire, Arousal, Lubrication, Orgasm, Satisfaction, and Pain. The domain scores were calculated by summing each item within the domain and multiplying them by the domain factor. Each domain score is summed to calculate the total score. The total scores range from 2 to 36 with higher scores indicating greater levels of sexual functioning.^46–48^ CICRC within the current sample was α = 0.97.

#### Endometriosis health profile-30

The EHP-30 is a 30-item measure used to assess HRQoL in individuals with endometriosis over a 5-point Likert scale ranging from 1 (never) to 5 (always). This scale score is equal to the total of the raw scores of each item divided by the maximum possible raw score of all items in the scale, multiplied by 100. This results in a scale score ranging from 0 (best health status) to 100 (worst health status).^7,49^ CICRC within the current sample was α = 0.97.

#### Visual analog scale

The visual analog scale (VAS) measured pain severity, specifically, “the level of pain caused by endometriosis.” The VAS consists of a 10-cm line with two endpoints representing 0 (no pain) and 10 (pain as bad as it could possibly be).^50,51^ Previous research identified the VAS as an instrument with good validity and excellent reliability, when assessing pain and quality of life.^52^

### Procedure

Following approval by the RMIT University Human Research Ethics Committee (HREC# 27655), the questionnaires were uploaded to a secure Qualtrics server. Participants were recruited through advertisements on Facebook and Reddit, alongside flyers in health clinics based in Melbourne, Australia. A website link or QR code was provided which directed participants to the Participant Information and Consent Form. Inclusion criteria were that participants are over the age of 18, fluent in English, and have received a diagnosis of endometriosis from a medical professional. This outlined the purpose of the study, what participation would involve, potential risks, and relevant ethical guidelines and rights including confidentiality and voluntary participation. After participants confirmed they had read the provided participant information sheet, they clicked the “Continue” on the Qualtrics button as written expressed informed consent. The questionnaire took approximately 25 min to complete. Following the survey, participants were invited to enter a draw to win one of three $50 vouchers, this was collected using a separate questionnaire to ensure that data remained unidentifiable.

### Data analysis

Data were screened and analyzed using statistics package IBM SPSS Statistics (Version 29; IBM Corp). Variables were initially screened for outliers and missing data. Prior to analysis, variables were assessed for normality, linearity, homoscedasticity, and multicollinearity. Correlational analysis was conducted to assess the relationship between all continuous variables and to assess hypothesis I. To assess hypothesis II and hypothesis III, multiple linear regression was conducted alongside a moderation analysis using the Hayes^53^ PROCESS mediation and moderation script, exploring the moderating effect of hypervigilance, pain catastrophizing and sexual functioning (hypothesis II), and the moderating effect of self-efficacy, functionality appreciation, hypervigilance, pain catastrophizing, and sexual functioning on pain and HRQoL (hypothesis III).

## Results

### Preliminary analysis

The Shapiro–Wilk test of normality identified several normality violations (*p* < 0.001); however, data were not transformed as parametric tests have endured such violations in studies with high participant counts.^54^ A total of 332 participants (female = 94%, male = 2.7%, non-binary/third gender = 3.3%) aged between 18 and 57 (*M* = 31.14, SD = 7.93) participated within this study. A visual inspection of scatterplots demonstrated linearity and homoscedasticity in all relevant demographics. These demographics are displayed in Table 1.

### Relationship between self-efficacy and functionality appreciation

Pearson’s product moment correlation was conducted to explore the relationships between self-efficacy and functionality appreciation (Table 2). The analysis revealed a moderate, positive relationship between self-efficacy and functionality appreciation (*r* (332) = 0.58, *p* < 0.01).

**Table 2.** Correlational analysis between self-efficacy and functionality appreciation.

Variable	1.	2.
1. GSE	—	
2. FAS	0.584**	—

* *p* < 0.05. **p < 0.01.

GSE: general self-efficacy scale; FAS: functionality appreciation scale.

Additionally, Pearson’s product moment correlation was conducted to explore the relationships between self-efficacy, functionality appreciation, hypervigilance, pain catastrophizing, sexual functioning, HRQoL, and pain, and has been included in the Appendix A section.

### Demonstration of the fear-avoidance model

Assumptions were checked, and multicollinearity was not observed. For all continuous variables, the variance inflation factor was below 10, and the tolerance value was above 0.10. Normal P–P plots revealed that all residuals were normally distributed. Homoscedasticity was observed as no obvious patterns emerged in the scatter plots.

To test the second hypothesis, a hierarchical multiple regression analysis was conducted to assess whether hypervigilance moderates the relationship between sexual functioning and pain. In the first step, a hierarchical regression was conducted to determine how sexual avoidance and hypervigilance predicted pain. The overall model was statistically significant $R^{2}$ = 0.09, *F*(3,328) = 11.24, *p* < 0.01 (Table 3), accounting for 9.32% of the variance in pain intensity. Mean square error (MSE), representing the average residual variance, was 4.21 for the hypervigilance model and 3.89 for the pain catastrophizing model, indicating comparable levels of unexplained variance across models.

**Table 3.** Model summaries for the relationship between sexual functioning and pain.

Moderating variable	$R^{2}$ *R* ^2^	MSE	*F*(3,328)	*p*
Hypervigilance	0.09	4.21	11.24	<0.001
Pain catastrophizing	0.16	3.89	21.12	<0.001

MSE: Mean square error.

Next, the interaction term between hypervigilance and sexual avoidance was added to the regression model. There was a significant interaction between sexual functioning and hypervigilance on pain levels (Table 4; *b* = 0.002, bias-corrected confidence interval (BcCI) [−0.004, −0.000], *t* = −2.607, *p* < 0.01).

**Table 4.** Interaction summaries for the relationship between sexual functioning and pain.

Moderating variable	*b*	SE	*T*	95% CI	*p*
Hypervigilance	−0.002	0.001	−2.607	[−0.004, −0.001]	0.010**
Pain catastrophizing	−0.000	0.001	−0.165	[−0.002, 0.002]	0.870

* *p* < 0.05. **p < 0.01.

CI: confidence interval; SE: standard error.

**Figure 1.**
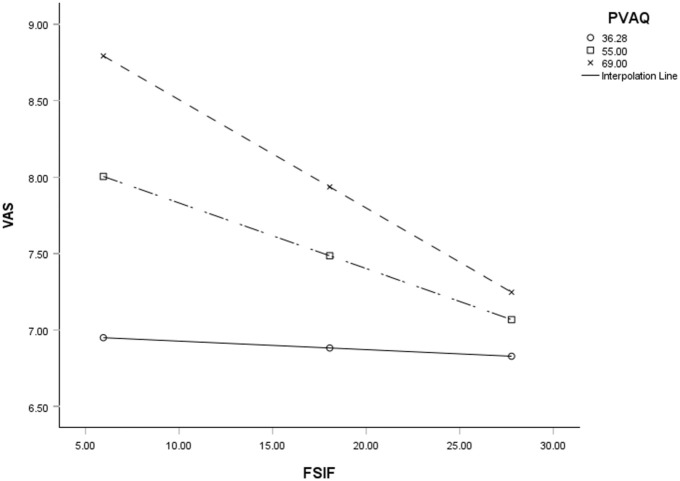
The moderation effect of hypervigilance on the relationship between sexual functioning and pain. This can be observed through each slope representing low (noted as ○), medium (noted as □), and high (noted as ×) levels of hypervigilance, and how it increases the relationship between high levels of pain and low sexual functioning. VAS = pain, FSFI = sexual functioning, PVAQ = hypervigilance. FSFI: female sexual function index; VAS: visual analog scale.

Examination of the interaction plot revealed an enhancing effect that as hypervigilance increased the negative relationship between sexual functioning and pain strengthened (Figure 1). This process was repeated to test whether pain catastrophizing moderates the relationship between sexual functioning and pain. The overall model was significant, $R^{2}$ = 0.16, *F*(3,328) = 21.12, *p* < 0.001, accounting for 16.2% of the variance in pain intensity. The interaction between these variables was not significant (*b* = −0.000, BcCI [−0.002, 0.002], *t* = −0.165, *p* = 0.870), indicating that the relationship between sexual functioning and pain is not moderated by pain catastrophizing.

This same method was utilized to test the third hypothesis. A hierarchical multiple regression analysis was conducted to determine whether self-efficacy, functionality appreciation, pain catastrophizing, or hypervigilance moderates the relationship between pain and HRQoL. The overall models were statistically significant, each accounting for a percentage of the variance; self-efficacy (43%), functionality appreciation (42.9%), hypervigilance (46.7%), pain catastrophizing (62.2%), and sexual functioning (42.3%; Table 5).

**Table 5.** Model summaries for the relationship between pain and HRQoL.

Moderating variable	$R^{2}$ *R*_2_	MSE	*F*(3,328)	*p*
Self-efficacy	0.43	170.01	82.55	<0.001
Functionality appreciation	0.43	170.13	82.42	<0.001
Hypervigilance	0.47	159.02	95.82	<0.001
Pain catastrophizing	0.62	112.66	180.25	<0.001
Sexual functioning	0.42	172.14	80.19	<0.001

HRQoL: health-related quality of life; MSE: Mean square error.

**Figure 2.**
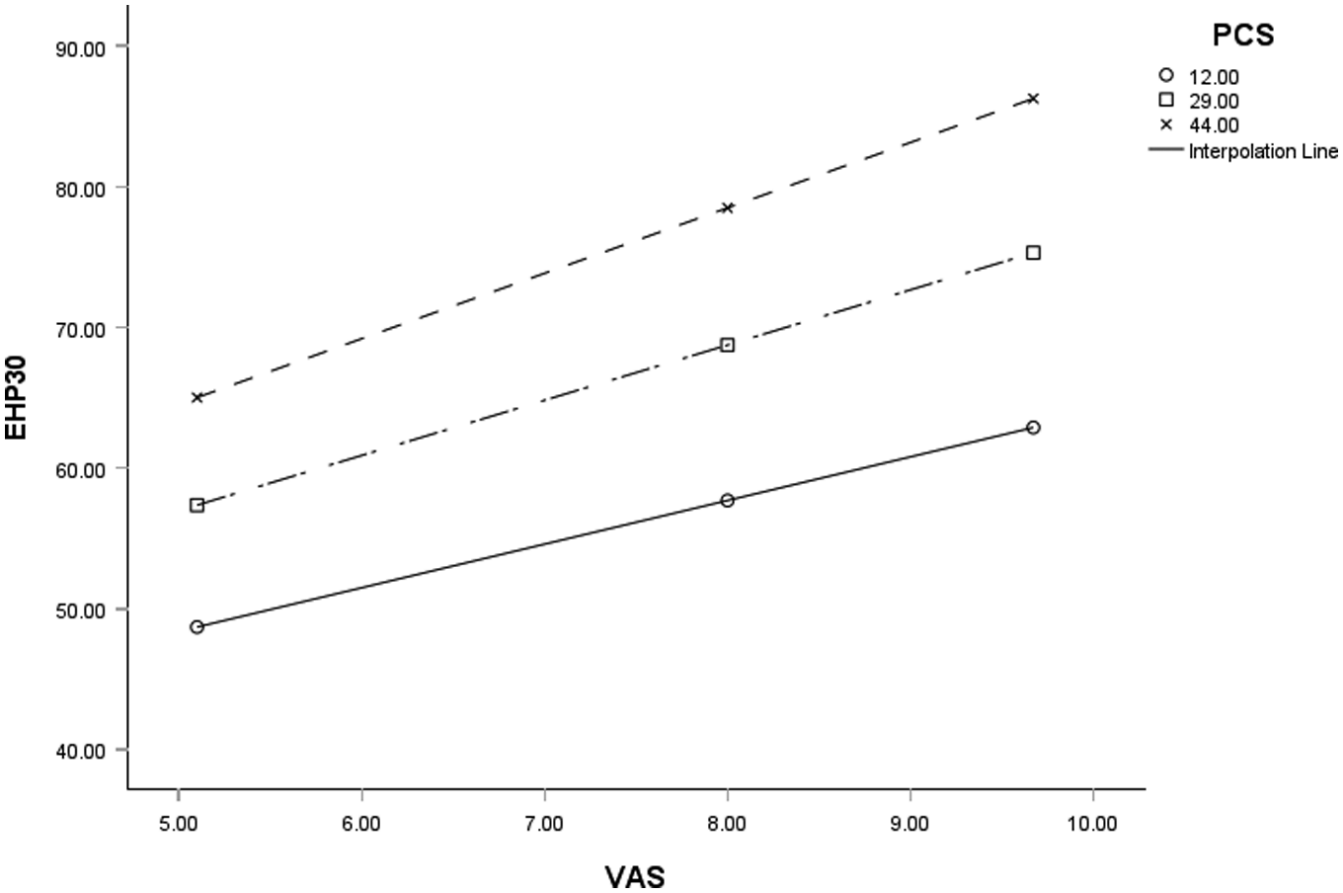
The moderation effect of pain catastrophizing on the relationship between pain and HRQoL. This can be observed through each slope representing low (noted as ○), medium (noted as □) and high (noted as ×) levels of pain catastrophizing, and how it increases the relationship between low HRQoL and high pain. EHP-30 = HRQoL, VAS = pain, PCS = pain catastrophizing. HRQoL: health-related quality of life; EHP-30: endometriosis health profile-30; PCS: pain catastrophizing scale; VAS: visual analog scale.

When testing the interaction between variables, pain catastrophizing was the only statistically significant moderator between pain and HRQoL (*b* = 0.048, BcCI [0.009, 0.087], *t* = 2.445, *p* = <0.05; Table 6). Examination of the interaction plot revealed an enhancing effect that as pain catastrophizing increased the negative relationship between pain and HRQoL strengthened (Figure 2).

**Table 6.** Interaction summaries for the relationship between pain and HRQoL.

Moderating variable	*b*	SE	*t*	95% CI	*p*
Self-efficacy	0.051	0.067	0.759	[−0.081, 0.182]	0.448
Functionality appreciation	0.763	0.390	1.958	[−0.004, 1.530]	0.051
Hypervigilance	0.023	0.020	1.182	[−0.016, 0.062]	0.238
Pain catastrophizing	0.048	0.020	2.445	[0.009, 0.087]	0.015*
Sexual functioning	0.010	0.033	0.291	[−0055, 0.075]	0.772

* *p* < 0.05. **p < 0.01.

HRQoL: health-related quality of life; CI: confidence interval; SE: standard error.

## Discussion

The current study aimed to explore the applications of the fear-avoidance model in individuals with endometriosis by assessing the relationships and interactions of variables associated with this model. As predicted by hypothesis I, functionality appreciation and self-efficacy were positively correlated. Hypothesis II was partially supported, as both regression models were significant predictors for pain; however, only hypervigilance demonstrated a moderating effect, enhancing the relationship between sexual functioning and pain indicating that although pain catastrophizing was a significant predictor of pain, it was not shown to have a moderating effect on the relationship between sexual functioning and pain. The final hypothesis was also partially supported, with each variable acting as a significant predictor of HRQoL; however, only pain catastrophizing demonstrated a moderating effect, enhancing the relationship between pain and HRQoL indicating that although self-efficacy, functionality appreciation, hypervigilance, and sexual functioning were all significant predictors of HRQoL, they did not have a moderating effect on the relationship between pain and HRQoL.

**Figure 3.**
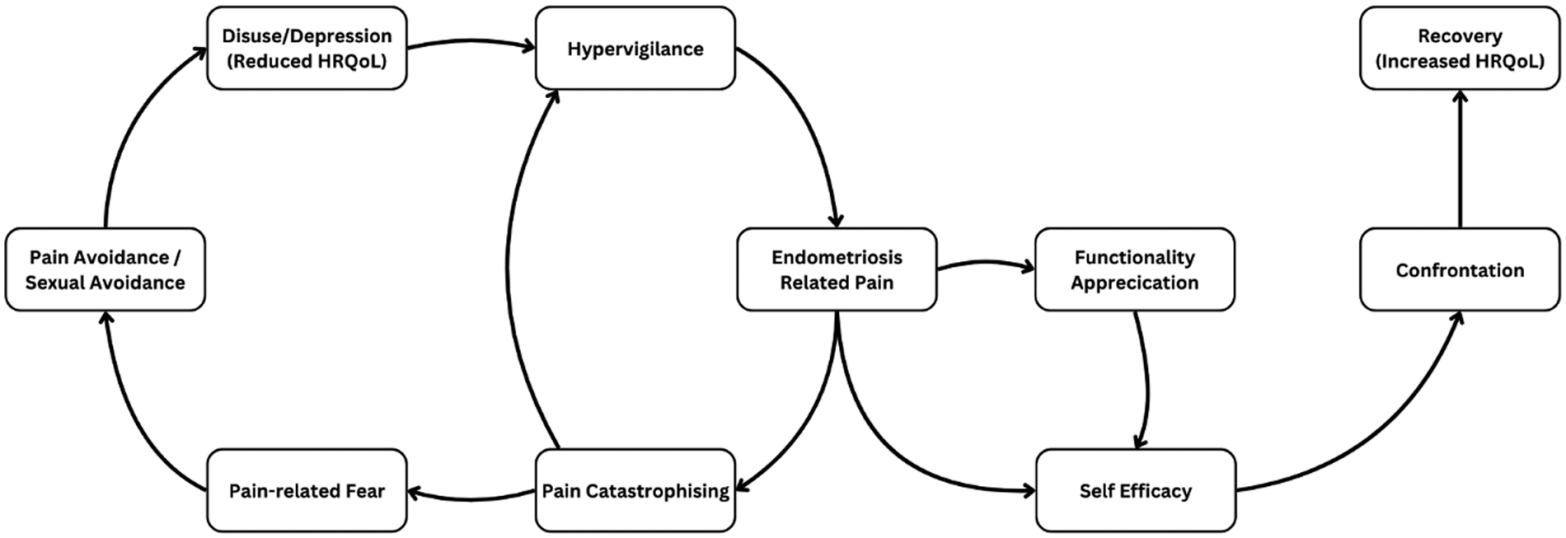
Proposed endometriosis-specific interpretation of the fear avoidance model.

These findings contribute to a more comprehensive understanding of the mechanisms underlying pain and HRQoL in people with endometriosis. The evidence indicates that the experience of pain in people with endometriosis functions similarly to the pain reported in other chronic pain conditions.^18^ Based on these results, Figure 3 was developed to present a graphical interpretation of the fear-avoidance model, incorporating the variables examined in the present study.

This model acts as a theoretical guide, describing the cascading events that follow the pain experience.^15^ Following previous iterations, this proposed model follows the cyclical events that lead to reduced HRQoL and increased pain intensity, in addition to following the pathway to recovery as characterized by engaging in previously avoided activities (noted as “Confrontation”), reduced pain and increased HRQoL.^28,37^

Notably, the relationship between sexual avoidance and pain severity is enhanced by high levels of hypervigilance and partially predicted by pain catastrophizing. This study aligns with previous research examining the application of the fear-avoidance model in other sexual pain populations, demonstrating that its application within endometriosis populations may provide a useful framework for informing future treatment pathways. Specifically, the fear-avoidance model may offer valuable insights for the development of interventions aimed at supporting sexual functioning and well-being in individuals with endometriosis.^21,55^

Only hypervigilance demonstrated a direct negative effect on the relationship between sexual avoidance and pain. This suggests that heightened attention, in this case hypervigilance, may play a critical important role in avoidance behaviors and the experience of pain in individuals with endometriosis.^19^ Typically, hypervigilance and pain catastrophizing are closely associated with chronic pain and sexual pain research.^18^ This association was considered during the development of the present study’s model, which incorporates hypervigilance as an important predictor of increased pain (Figure 3). Although pain catastrophizing showed greater predictive power, the precise nature of its interaction with sexual avoidance and pain requires additional exploration. In the context of endometriosis, it is proposed that pain catastrophizing may reinforce hypervigilance as expectations of heightened pain increase attention toward painful stimuli, caused by the multiple pain symptoms associated with endometriosis.^19^ As this proposed model is based on initial findings, further investigation to refine this model is needed, including examination of the interaction between hypervigilance and pain catastrophizing in endometriosis populations.

The exploration of protective factors within this study highlights the role of functionality appreciation and self-efficacy within the fear-avoidance model and individuals with endometriosis. Although these two variables did not affect the nature of the relationship between pain and HRQoL, both self-efficacy and functionality appreciation demonstrated comparative predictive power. These findings are consistent with previous research examining the potential applications of functionality appreciation and pain and sexual distress in endometriosis.^30,31^ Functionality appreciation remains an underexplored construct; however, the finding that functionality appreciation is correlated with reduced pain and increased HRQoL highlights its relevance for individuals with endometriosis.

The results of the present study are consistent with previous research identifying self-efficacy as a protective factor in the fear-avoidance model.^26,55–57^ O’Hara et al.^56^ examined self-management factors among women with endometriosis and found that higher levels of self-efficacy were associated with more effective pain management and greater social and economic participation, contributing to improved quality of life. In line with these findings, the present study found that higher self-efficacy was associated with lower pain levels and increased HRQoL. Together these studies demonstrate the protective role that self-efficacy plays in endometriosis.

Notably, functionality appreciation and self-efficacy demonstrated comparable effects in both correlational and hierarchical regression analyses. Markey et al.^30^ suggested that perceived ability to manage pain led to a greater sense of gratitude toward one’s body. Given that the perceived ability to overcome challenges is a fundamental aspect of self-efficacy, the relationship between functionality appreciation and self-efficacy was considered to have a “flow-on” effect with high levels of functionality appreciation leading toward increased self-efficacy (Figure 3). This interaction may represent a crucial step toward recovery. Previous research has also suggested that pain resilience plays a role within the fear-avoidance model.^28^ Although resilience was not included in the present analyses, future research would benefit from examining its relationship with self-efficacy and functionality appreciation to further elucidate the components of this model.

Overall, the proposed model demonstrates how functionality appreciation, self-efficacy, hypervigilance, pain catastrophizing, and sexual avoidance collectively contribute to the relationship between pain and HRQoL. As endometriosis is a chronic pain condition, “Recovery” was conceptualized as increased HRQoL, whereas “Depression and Disuse” represented the reduction in HRQoL caused by the fear-avoidance cycle (Figure 3).

Self-efficacy, functionality appreciation, hypervigilance, and sexual avoidance each displayed comparative predictive power in regression analysis (43%, 43%, 46%, and 42%, respectively). Pain catastrophizing, however, demonstrated higher predictive power (62%) and was also shown to have a direct effect on the relationship between pain and HRQoL. These findings are consistent with previous research in both endometriosis and broader chronic pain populations, emphasizing the contribution of increased pain to reduced HRQoL and the amplifying role of pain catastrophizing in this relationship.^3,35^ Previous research has shown that pain catastrophizing has a mediating effect on the relationship between pain and HRQoL.^35^ The present findings extend this literature by reinforcing the central role of pain catastrophizing within the proposed model. In line with existing evidence, these results support the development of interventions targeting pain catastrophizing as valuable strategies for pain management and improving HRQoL among individuals with endometriosis.

### Limitations

This study has a number of limitations. Foremost, the present study was correlational in design; therefore, causality cannot be inferred. This study also collected participants and data online, including within endometriosis support groups, highlighting a potential selection bias. Additionally, there was no adjustment for potential confounding variables, such as age or hormonal contraceptive use. These variables could have affected the relationship observed within this study.

Diagnosis of endometriosis has been reported to be delayed by 6.6 years, meaning this study does not capture the length of time a participant had been experiencing symptoms of endometriosis, which may have influenced the results of this study.^1,33,58^ Similarly, this study did not specify the type of pain experienced that was caused by the participant’s endometriosis such as dysmenorrhea, dyspareunia, or non-cyclical pelvic pain. The latter of which is considered unpredictable and may have a different relationship between the variables studied compared to more predictable pain caused by endometriosis.

The present study focused primarily on sexual avoidance behaviors during data collection and analysis utilizing the FSFI as the sexual avoidance measure. While this method reflected the common symptoms associated with endometriosis, it does not reflect potential day-to-day avoidance that may occur and contribute to the fear-avoidance model such as avoiding exercise or physical therapy, both of which are considered important for relieving pain symptoms.^3,59^ Further research reviewing alternative avoidance behaviors and measures would be valuable when looking into the fear-avoidance model’s applications and understanding the psychological components that contribute to pain and HRQoL in people with endometriosis.

Similarly, this study focused on each variable of the fear-avoidance model separately. The fear-avoidance model is described as an interaction of multiple variables; therefore, there may be nuance in the contribution of self-efficacy, functionality appreciation, hypervigilance, and pain catastrophizing as a whole that is yet to be observed.^15^ Future research should aim to develop and refine this model in order to adapt it to clinical interventions for individuals experiencing chronic pain caused by endometriosis.

### Clinical implications

This study provides evidence of the fear-avoidance model’s application in populations with endometriosis and informs areas of further research to refine the theoretical groundwork of the psychological factors that contribute to pain and HRQoL in those with endometriosis.

In the absence of a cure for endometriosis, approaches for managing and reducing pain symptoms and distress are imperative.^60^ Elements such as self-efficacy can be bolstered through education and behavioral interventions such as graded activity and graded exposure.^26^ While alternatively, a reduction of pain catastrophizing and hypervigilance may reduce pain intensity and in turn increase HRQoL. With this knowledge, clinicians would be equipped with the skills to support individuals with endometriosis, not only assist with the distress caused by the experience of living with a chronic gynecological condition but to also reframe the cognitive and behavioral patterns that exacerbate the condition. In turn, this research informs the development of specific and effective psychological interventions.

## Conclusion

The overall findings of the present study revealed the potential role functionality appreciation shares with self-efficacy as a protective factor against increased pain in endometriosis. Hypervigilance was shown to moderate the relationship between sexual avoidance and pain by drawing attention to painful stimuli, thus increasing pain intensity. Pain catastrophizing, on the other hand, moderated the relationship between pain and HRQoL, further highlighting the role the fear-avoidance model has in the development and exacerbation of chronic pain conditions.

Further research should address the refinement of the fear-avoidance model surrounding alternative avoidance behaviors, and the interactions between each variable. Ultimately, these results emphasize the need for endometriosis to be treated with the seriousness of a prevalent chronic pain condition with complex elements that requires further research to prepare clinicians and therapists to treat those with endometriosis effectively.

## Supplemental Material

sj-pdf-1-whe-10.1177_17455057261444146 – Supplemental material for The relationship between psychological factors and pain in endometriosis Supplemental material, sj-pdf-1-whe-10.1177_17455057261444146 for The relationship between psychological factors and pain in endometriosis by R. Favaloro, K. T. Hallam and P. Saunders in Women's Health

## Appendix

**Appendix A.** Test of normality.

Variable	Shapiro–Wilk statistic	*df*	*p*
GSE	0.982	332	<0.001*
FAS	0.951	332	<0.001*
PVAQ	0.963	332	<0.001*
PCS	0.969	332	<0.001*
FSFI	0.963	332	<0.001*
EHP-30	0.972	332	<0.001*
VAS	0.915	332	<0.001*

EHP-30: endometriosis health profile-30; FSFI: female sexual function index; GSE: general self-efficacy scale; FAS: functionality appreciation scale; PVAQ: pain vigilance and awareness questionnaire; PCS: pain catastrophizing scale; VAS: visual analog scale.

* Assumption was violated.

**Table:** Correlational analysis between scales.

Variable	1.	2.	3.	4.	5.	6.	7.
1. GSE	—						
2. FAS	0.584**	—					
3. PVAQ	0.059	0.172**	—				
4. PCS	−0.098	−0.040	0.614**	—			
5. FSFI	0.282**	0.313**	−0.087	−0.117*	—		
6. EHP-30	−0.203**	−0.233**	0.369**	0.662**	−0.218**	—	
7. VAS	−0.109*	−0.198**	0.224**	0.380**	−0.174**	0.641**	—

GSE: general self-efficacy scale; FAS: functionality appreciation scale; PVAQ: pain vigilance and awareness questionnaire; PCS: pain catastrophizing scale; FSFI: female sexual function index; EHP-30: endometriosis health profile-30; VAS: visual analog scale.

* *p* < 0.05. ***p* < 0.01.

**Table:** Missing data prior to multiple imputation.

	Missing	
Variable	Count	Percentage
GSE		
Item 1	1	0.3
Item 2	1	0.3
Item 3	1	0.3
Item 4	1	0.3
Item 5	0	0.0
Item 6	0	0.0
Item 7	0	0.0
Item 8	0	0.0
Item 9	0	0.0
Item 10	2	0.6
FAS		
Item 1	4	1.2
Item 2	1	0.3
Item 3	1	0.3
Item 4	0	0.0
Item 5	0	0.0
Item 6	1	0.3
Item 7	0	0.0
PVAQ		
Item 1	0	0.0
Item 2	1	0.3
Item 3	1	0.3
Item 4	0	0.0
Item 5	0	0.0
Item 6	1	0.3
Item 7	2	0.6
Item 8	0	0.0
Item 9	0	0.0
Item 10	0	0.0
Item 11	1	0.3
Item 12	0	0.0
Item 13	1	0.3
Item 14	0	0.0
Item 15	0	0.0
Item 16	4	1.2
PCS		
Item 1	1	0.3
Item 2	1	0.3
Item 3	2	0.6
Item 4	0	0.0
Item 5	0	0.0
Item 6	0	0.0
Item 7	2	0.6
Item 8	0	0.0
Item 9	2	0.6
Item 10	0	0.0
Item 11	1	0.3
Item 12	0	0.0
Item 13	1	0.3
FSFI		
Item 1	2	0.6
Item 2	1	0.3
Item 3	1	0.3
Item 4	0	0.0
Item 5	0	0.0
Item 6	0	0.0
Item 7	0	0.0
Item 8	1	0.3
Item 9	1	0.3
Item 10	3	0.9
Item 11	4	1.2
Item 12	3	0.9
Item 13	2	0.6
Item 14	10	3.0
Item 15	13	3.9
Item 16	9	2.7
Item 17	4	1.2
Item 18	3	0.9
Item 19	3	0.9
EHP-30		
Item 1	3	0.9
Item 2	3	0.9
Item 3	3	0.9
Item 4	2	0.6
Item 5	2	0.6
Item 6	3	0.9
Item 7	2	0.6
Item 8	3	0.9
Item 9	1	0.3
Item 10	2	0.6
Item 11	2	0.6
Item 12	2	0.6
Item 13	1	0.3
Item 14	1	0.3
Item 15	1	0.3
Item 16	1	0.3
Item 17	2	0.6
Item 18	3	0.9
Item 19	1	0.3
Item 20	2	0.6
Item 21	2	0.6
Item 22	1	0.3
Item 23	1	0.3
Item 24	3	0.9
Item 25	1	0.3
Item 26	2	0.6
Item 27	1	0.3
Item 28	2	0.6
Item 29	2	0.6
Item 30	2	0.6
VAS		
Item 1	9	2.7

EHP-30: endometriosis health profile-30; FSFI: female sexual function index; GSE: general self-efficacy scale; FAS: functionality appreciation scale; PVAQ: pain vigilance and awareness questionnaire; PCS: pain catastrophizing scale; VAS: visual analog scale.
