# Supplementary material for: The relationship between psychological factors and pain in endometriosis
Source: Womens Health (Lond). 2026 Apr 27;22:17455057261444146. doi: 10.1177/17455057261444146 (PMC13133433; doi:10.1177/17455057261444146)
Supplement: sj-pdf-1-whe-10.1177_17455057261444146 – Supplemental material for The relationship between psychological factors and pain in endometriosis [file sj-pdf-1-whe-10.1177_17455057261444146.pdf]

Strobe Checklist (von Elm et al., 2007)

	Item Number	Recommendation	Page Number
**Title and Abstract**	1	(a) Indicate the study's design with a commonly used term in the title or the abstract<br>(b) Provide in the abstract an informative and balanced summary of what was done and what was found	1-3
Background and Rationale	2	Explain the scientific background and rationale for the investigation being reported	4-9
Objectives	3	State specific objectives, including any prespecified hypotheses	9
**Methods**			
Study Design	4	Present key elements of study design early in the paper	1, 9
Setting	5	Describe the setting, locations, and relevant dates, including periods of recruitment, exposure, follow-up, and data collection	9-10
Participants	6	(a) Give the eligibility criteria, and the sources and methods of selection of participants	9-10
Variables	7	Clearly define all outcomes, exposures, predictors, potential confounders, and effect modifiers. Give diagnostic criteria, if applicable	10-13
Data Sources	8	For each variable of interest, give sources of data and details of methods of assessment (measurement). Describe comparability of assessment methods if there is more than one group	11-13
Bias	9	Describe any efforts to address potential sources of bias	25
Study Size	10	Explain how the study size was arrived at	10
Quantitative Variables	11	Explain how quantitative variables were handled in the analyses. If applicable, describe which groupings were chosen and why	10-13
Statistical Method	12	(a) Describe all statistical methods, including those used to control for confounding<br>(b) Describe any methods used to examine subgroups and interactions	10, 13, 14

		(c) Explain how missing data were addressed<br>(d) If applicable, describe analytical methods taking account of sampling strategy<br>(e) Describe any sensitivity analyses	
**Results**			
Participants	13	(a) Report numbers of individuals at each stage of study—eg numbers potentially eligible, examined for eligibility, confirmed eligible, included in the study, completing follow-up, and analyzed<br>(b) Give reasons for non-participation at each stage<br>(c) Consider use of a flow diagram	10
Descriptive Data	14	(a) Give characteristics of study participants (eg demographic, clinical, social) and information on exposures and potential confounders<br>(b) Indicate number of participants with missing data for each variable of interest	14
Outcome Data	15	Report numbers of outcome events or summary measures	15-17
Main Results	16	(a) Give unadjusted estimates and, if applicable, confounder-adjusted estimates and their precision (eg, 95% confidence interval). Make clear which confounders were adjusted for and why they were included<br>(b) Report category boundaries when continuous variables were categorized<br>(c) If relevant, consider translating estimates of relative risk into absolute risk for a meaningful time period	15-17
Other Analyses	17	Report other analyses done—eg analyses of subgroups and interactions, and sensitivity analyses	NA
**Discussion**			
Key Results	18	Summarize key results with reference to study objectives	17
Limitations	19	Discuss limitations of the study, taking into account sources of potential bias or imprecision. Discuss both direction and magnitude of any potential bias	21-23
Interpretation	20	Give a cautious overall interpretation of results considering objectives, limitations,	18-21

		multiplicity of analyses, results from similar studies, and other relevant evidence	
**Generalizability**	21	Discuss the generalizability (external validity) of the study results	20-23
**Other information**			
Funding	22	Give the source of funding and the role of the funders for the present study and, if applicable, for the original study on which the present article is based	25

von Elm, E., Altman, D. G., Egger, M., Pocock, S. J., Gøtzsche, P. C., & Vandenbroucke, J. P. (2007). The Strengthening the Reporting of Observational Studies in Epidemiology (STROBE) statement: guidelines for reporting observational studies. *Ann Intern Med*, *147*(8), 573-577. https://doi.org/10.7326/0003-4819-147-8-200710160-00010
